# Development of Non-Ethoxypropanoic Acid Type Cryptochrome Inhibitors with Circadian Molecular Clock-Enhancing Activity by Bioisosteric Replacement

**DOI:** 10.3390/ph14060496

**Published:** 2021-05-24

**Authors:** Yong Uk Jeong, Hyo-Eon Jin, Hye Young Lim, Goyeong Choi, Hansol Joo, Bohun Kang, Ga-Hyun Lee, Kwang-Hyeon Liu, Han-Joo Maeng, Sooyoung Chung, Gi Hoon Son, Jong-Wha Jung

**Affiliations:** 1 Research Institute of Pharmaceutical Sciences, College of Pharmacy, Kyungpook National University, Daegu 41566, Korea; nemo946@naver.com (Y.U.J.); qlgudtmfkqmf@naver.com (G.C.); wnthf0518@naver.com (H.J.); bohun0609@gmail.com (B.K.); leegh2710@naver.com (G.-H.L.); dstlkh@knu.ac.kr (K.-H.L.); 2 Vessel-Organ Interaction Research Center, Kyungpook National University, Daegu 41566, Korea; 3 College of Pharmacy, Ajou University, Suwon 16499, Korea; hjin@ajou.ac.kr; 4 Department of Biomedical Sciences, College of Medicine, Korea University, Seoul 02841, Korea; yeppi0905@naver.com; 5 College of Pharmacy, Gachon University, Incheon 21936, Korea; hjmaeng@gachon.ac.kr; 6 Department of Brain and Cognitive Sciences, Scranton College, Ewha Womans University, Seoul 03760, Korea; csooy@ewha.ac.kr

**Keywords:** circadian rhythm, circadian clock, cryptochrome inhibitor, bioisosteric replacement

## Abstract

Circadian dysfunction is closely associated with an increased risk of various diseases. Considering that molecular clock machinery serves as an intrinsic time-keeping system underlying the circadian rhythm of biological processes, the modulation of the molecular clock machinery is an attractive therapeutic target with novel mechanisms of action. Based on the previous structure–activity relationship study of small molecule cryptochrome (CRY) inhibitors possessing an ethoxypropanoic acid moiety, non-ethoxypropanoic acid-type inhibitors have been developed by bioisosteric replacement. They were evaluated as potent and effective enhancers of E-box-mediated transcription, and, in particular, ester **5d** and its hydrolysis product **2d** exhibited desirable metabolic and pharmacokinetic profiles as promising drug candidates. Compound **2d** directly bound to both CRY1 and 2 in surface plasmon resonance analyses, suggesting that the molecular target is CRY. Effects of compound **5d** and **2d** on suppressive action of CRY1 on CLOCK:BMAL1-activated E-box-LUC reporter activity revealed that both compounds inhibited the negative feedback actions of CRY on CLOCK:BMAL1. Most importantly, compounds **5d** and **2d** exhibited significant effects on molecular circadian rhythmicity to be considered circadian clock-enhancers, distinct from the previously developed CRY inhibitors possessing an ethoxypropanoic acid moiety.

## 1. Introduction

Circadian rhythms of biological processes are evolutionarily well-conserved and ubiquitous biological oscillations with an approximate 24 h daily cycle. The rhythm produced by the intrinsic time-keeping system coordinates internal biological processes to anticipate periodic environmental changes. The intrinsic time-keeping system, also known as the circadian clock, is closely related to behavioral, physiological, and metabolic cyclicities. Numerous studies have revealed that the increased risk of various common diseases is associated with circadian dysfunctions, which are attributed to environmental disruptions and intrinsic misalignment [1]. Thus, the circadian clock is attracting attention as a potential therapeutic target with an improved understanding of the circadian basis of health and disease. Behavioral intervention [2], chronotherapeutic drug administration and delivery, and the modulation of the molecular clock components improved existing therapies and developed novel ones [3]. Moreover, infections are also closely related to circadian rhythms [4]. It was recently reported that COVID-19 management is significantly affected by the circadian clock, which controls the immune system, virus replication, and infection severity [5].

Therefore, attempts for chemical regulation of the circadian clock system have emerged; small molecule modulators of core clock components have also been recently developed. For example, KL001 is the first-in-class activator of cryptochrome (CRY) [6]. Derivatives of KL001 have also been extensively explored; for example, CRY1/2 isoform-selective activators, KL101 and TH301, along with their derivatives were recently reported [7], and improved CRY1-selective stabilizers such as TH303 and TH129 were also developed [8]. More importantly, several azobenzene derivatives of TH129 were developed as CRY1-selective and light-controllable stabilizers, enabling the spatiotemporal manipulation of the circadian clock [8]. Besides, small molecules and natural products for the other core clock components have been identified and developed. GSK4112 is the first synthetic NR1D1/2 agonist [9], and the optimization of GSK4112 provided various agonists and antagonists for NR1D1/2. Cholesterol and its analogs are natural ligands of ROR or ROR. Stearic and retinoic acids are natural ligands of ROR. Natural products, such as neoruscogenin [10] and nobiletin [11] were also reported as ROR ligands. T0901317 [12] and its derivatives are representative synthetic modulators targeting RORs. Reviews are available on the development of small molecule modulators targeting molecular clock components [13,14,15].

**Figure 1:**
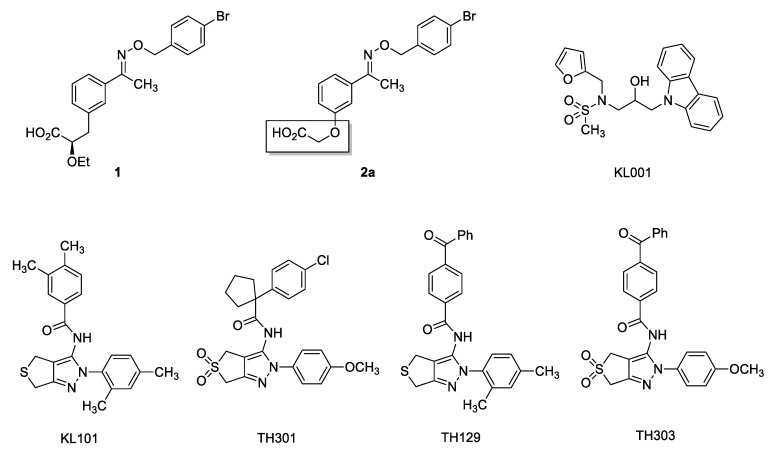
The chemical structure of KS15 (**1**), SJ019 (**2a**), and selected CRYs modulators.

KS15 (**1**, Figure 1), an ethoxypropanoic acid derivative, and its analogs, are small molecules that target CRY in the mammalian clock machinery [16]. To the best of our knowledge, KS15 is the first synthetic CRY inhibitor. KS15 augmented E-box-mediated transcription. KS15 directly binds to both CRY1 and CRY2, and attenuated rhythmic transcription of clock-controlled genes in cultured fibroblasts in a CRY1/2-dependent fashion. It has also been suggested that inhibition of CRY by KS15 exerts antiproliferative effects on human breast cancer cell cultures [17]. A recent mode-of-action study revealed that KS15 inhibited the interaction between CRYs and brain and muscle Arnt-like Protein 1 (BMAL1), thereby impairing CRY-evoked negative feedback actions on E-box-dependent transcription by CLOCK:BMAL1 heterodimer [18].

During the structure–activity relationship study of KS15 analogs, bioisosteric replacement of the ethoxypropanoic acid moiety provided a non-ethoxypropanoic acid-type KS15 analog, SJ019 (**2a**, Figure 1) [18]. SJ019 exhibited a comparable effect with KS15 on E-box-mediated transcription and circadian oscillations of the Bmal1 promoter. Interested in the non-ethoxypropanoic acid-type KS15 analog, we undertook further optimization by the structural tuning and evaluation of pharmacological and pharmacokinetic profiles, expecting the possibility of achieving optimal drug-like profiles. Here, we report the pharmacological and pharmacokinetic assessments of the non-ethoxypropanoic acid-type KS15 analogs for cryptochrome inhibitor development.

## 2. Results and Discussion

**Scheme 1:**
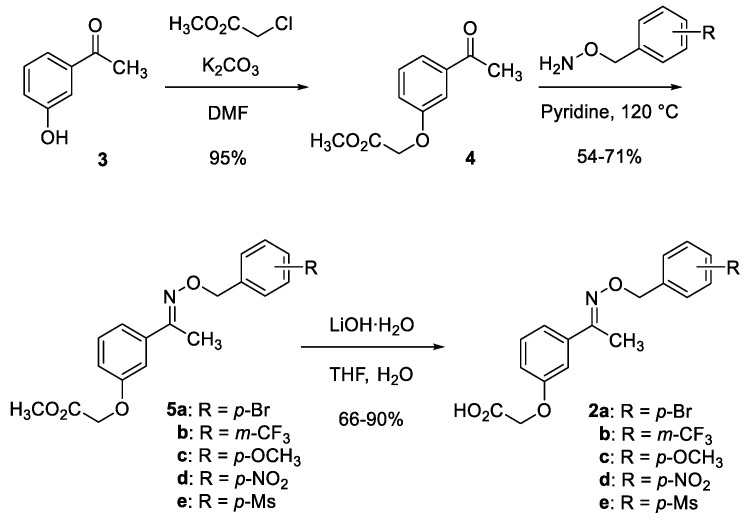
Synthesis of non-ethoxypropanoic acid type KS15 analogs.

Our work commenced with the synthesis of non-ethoxypropanoic acid-type SJ019 analogs. As shown in Scheme 1, the analogs were synthesized in a similar way to SJ019 [18]. Briefly, commercially available 3-hydroxyacetophenone 3 was alkylated to possess an oxypropanoic acid moiety as a bioisostere of the 2-ethoxypropanoic acid moiety of KS15. Then, the resulting acetophenone 4 was condensed with variously substituted benzyloxyamines to afford oximes **5a**–**e**. Hydrolysis of **5a**–**e** possessing terminal ester provided carboxylic acid **2a**–**e**.

With the available selected compounds, their effects on the molecular circadian rhythms were examined. As shown in Table 1, carboxylic acids **2a**–**e** and their methyl ester **5a**–**e** were examined with an expectation of developing prodrugs, but they showed similar potency and efficacy. Following the previous structure–activity relationship study, most of the analogs were potent transcriptional enhancers, except for **2e** and **5e** with the *para*-methylsulfonyl group. Among the analogs, those with the *para*-methoxy (**2c** and **5c**) and the *para*-nitro groups (**2d** and **5d**) were more effective transcriptional enhancers compared to SJ019 (**2a**). Notably, KS15 itself with the *para*-bromo group was the most potent and effective transcriptional enhancer among the KS15 derivatives possessing the 2-ethoxypropanoic acid moiety. Thus, a combination of functional groups and chemical moieties in the analogs tune the transcriptional activities. Finally, compound **5d** was chosen for further development in terms of efficacy and potency.

To further develop the selected compound **5d**, inhibitory activities of **5d** against five cytochrome P450 (P450) isozymes were examined using a cocktail method with human liver microsomes (HLMs) and P450 probe drugs. KS15 exhibited moderate inhibitory activities against CYP1A2 (IC_50_ = 14.9 μM), CYP2C9 (IC_50_ = 9.1 μM), CYP2C19 (IC_50_ = 20.2 μM), CYP2D6 (IC_50_ = 23.1 μM), and CYP3A (IC_50_ = 24.0 μM) (Table 2). Resultantly, KS15 has raised the potential of drug–drug interaction through metabolism. In contrast, the selected analog **5d** did not significantly interfere with the P450 isozymes at the tested concentrations, and all the IC_50_ values were more than 50 μM. Moreover, metabolites from the incubation of **5d** with HLMs during the experiments did not significantly interfere with the P450 isozymes.

**Figure 2:**
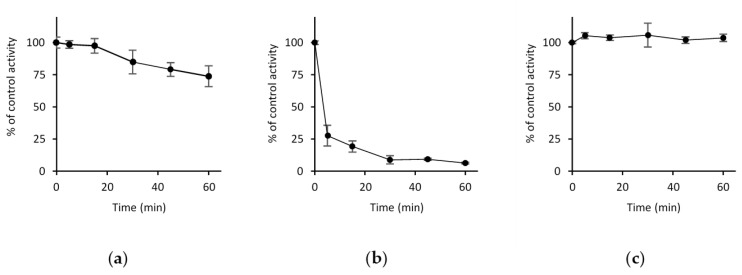
Metabolic stability of KS15 (**a**), **5d** (**b**), and **2d** (**c**) in human liver microsomes in the presence of an NADPH generating system. Data are the means ± SD of triplicate experiments.

Next, we examined the metabolic stability of **5d** in HLMs. Rapid hydrolysis of **5d** was anticipated because **5d** possesses a methyl ester group in its chemical structure. As shown in Figure 2, KS15 was relatively stable (t_1/2_ > 60 min), while **5d** was time-dependently decreased in HLMs (t_1/2_ = 2.4 min). The metabolite was determined as the hydrolysis product, i.e., **2d**, by mass spectrometry. We also examined the metabolic stability of **2d**, which was found to be stable in HLMs (t_1/2_ > 60 min).

**Figure 3:**
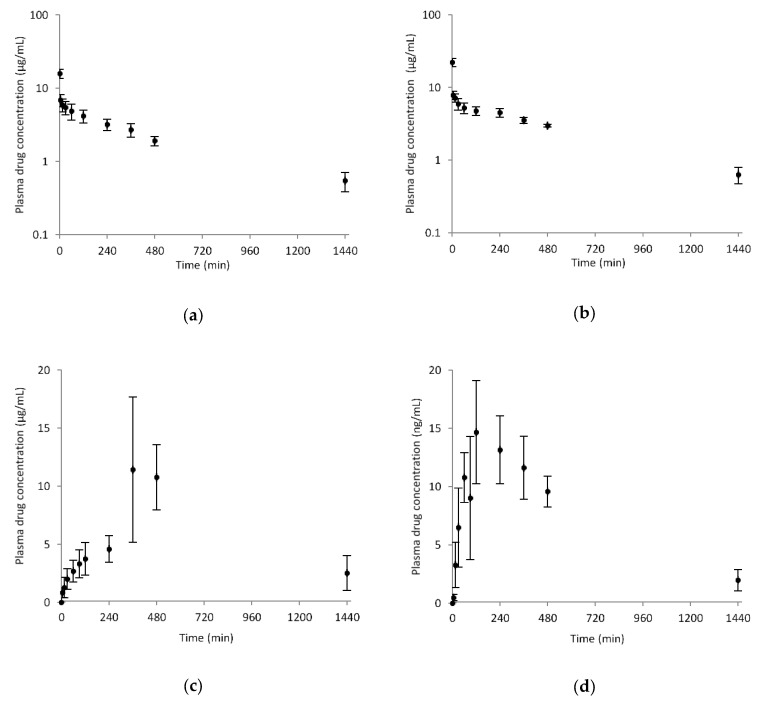
Pharmacokinetic profiles of **5d** and **2d**: Plasma concentration vs. time profiles after IV administration of 2 mg/kg of (**a**) **5d** and (**b**) **2d** to rats (mean ± SD, n = 3–4). Mean plasma concentration profiles after single PO administration of 5 mg/kg of (**c**) **5d** and (**d**) **2d** in rats (mean ± SD, n = 3–4).

We also conducted pharmacokinetic studies in vivo in rats after intravenous (IV, 2 mg/kg) or an oral (PO, 5 mg/kg) administration of **5d** and **2d** (Figure 3). For the pharmacokinetic study of **5d**, the concentrations of **2d** (formed metabolite) in plasma were measured after the IV or PO administration of **5d**, due to its metabolic instability. Pharmacokinetic parameters following IV or PO administration of each compound were summarized for the **2d** form in Table 3.

When we compared the pharmacokinetic characteristics of **5d** with that of **2d** (Figure 3), pharmacokinetic profiles after IV administrations of each compound were similar to what was expected. Table 3 shows some pharmacokinetic differences: AUCs after IV administration of **5d** were smaller than those of **2d**, while Vss was slightly larger. We speculated that those differences might be attributed to that the fact that the hydrolysis of **5d** to **2d** is fast but not prompt in vivo, and which is supported by slightly bigger values of **5d** in half-life (t_1/2_), clearance (CL), and mean retention time (MRT). Interestingly, the total clearance was slow as the **2d** form and Vss was small, indicating the limited tissue distribution of the **2d** form in the body. For PO administrations, the absorptions were almost complete (i.e., approximately 100%) in both cases, but a significant delay in t_max_ was observed for **5d**. Both **5d** and **2d** might be super-permeable from the gastrointestinal tract to the systemic blood circulation with a negligible first-pass effect on the **2d**. Otherwise, **5d** might be rapidly converted to a super-permeable **2d**, absorbed in the gastrointestinal tract. Indeed, the C_max_ values were high (i.e., 13.5 and 14.6 μg/mL for **5d** and **2d** administration). The T_max_ for PO administration of **5d** was prolonged with a shift of the curve compared with the PO administration of **2d**, possibly due to a delay in absorption or due to a metabolic conversion.

**Figure 4:**
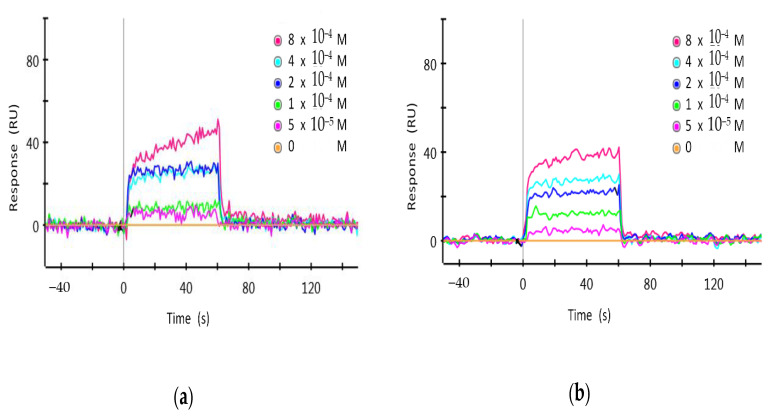
Surface plasmon resonance (SPR) analysis for the interaction between **2d** and purified CRY1 or CRY2 proteins: His-tagged CRY1 and CRY2 proteins were purified and used for SPR analysis. RU = resonance units. (**a**) Interaction between **2d** and CRY1. K_D_ = 3.89 × 10^−4^ M. (**b**) Interaction between **2d** and CRY2. K_D_ = 3.80 × 10^−4^ M.

In the next set of experiments, we examined whether compound **2d** acts on mammalian CRYs. Therefore, direct binding between compound **2d** and CRY1/2 was analyzed by surface plasmon resonance. Previously, biotin-tagged KS15 was reported to directly bind to the C-termini of both CRY1 and 2 in a pull-down assay [15]. Co-immunoprecipitation experiments also suggested that the binding of KS15 to CRY inhibits the interaction between CRY and BMAL1, thereby disrupting the feedback action of a circadian transcription-repressor complex [17]. As shown in Figure 4, compound **2d** is directly bound to CRY1 and CRY2 to a similar extent, indicating that the molecular target of **2d** is CRY1/2 for the modulation of the circadian clock.

**Figure 5:**
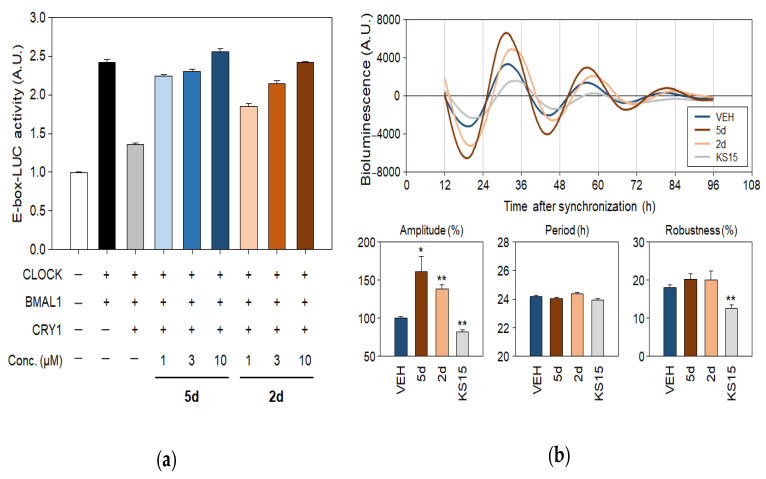
The circadian molecular clock-enhancing activity of **5d** and **2d**. (**a**) Effects of **5d** and **2d** on suppressive action of CRY1 on the CLOCK:BMAL1-activated E-box-LUC reporter activity. (**b**) Bioluminescence recordings of dexamethasone (DEX)-synchronized Per2-luciferase knock-in (Per2-Luc-KI) fibroblast cultures. Representative traces for rhythmic Per2-Luc activity (upper) and Cosinor analysis results (lower panels; mean ± SE, n = 4). * *p* < 0.05; one-way ANOVA with Newman-Keuls multiple comparison test for post-hoc comparisons; ** *p* < 0.01; one-way ANOVA with Newman-Keuls multiple comparison test for post-hoc comparisons.

In good agreement with the binding assay, compounds **5d** and **2d** functionally affected the negative feedback actions of CRY on the CLOCK:BMAL1 heterodimer. As shown in Figure 5a, enhanced E-box-mediated transcription by the CLOCK:BMAL1 heterodimer is strongly suppressed by co-expression of human CRY1. However, cell treatment with the compounds **5d** or **2d** dose-dependently restored E-box-mediated transcriptional activities, demonstrating their inhibitory effects on CRY-mediated negative feedback actions.

Most importantly, **5d** and **2d** are distinct from ethoxypropanoic acid-type KS15 regarding their effects on molecular circadian rhythmicity measured by periodic PER2 protein accumulation. Following our previous results [16], treatment with 20 μM KS15 attenuates amplitude but did not significantly alter the period of the rhythm in dexamethasone (DEX)-synchronized Per2-luciferase knock-in (Per2-Luc-KI) fibroblast cultures (Figure 5b). However, **5d** and **2d** (20 μM each) significantly augmented the amplitude of cyclic Per2 expression. Furthermore, they barely affect the robustness of the rhythm as examined by the Cosinor regression, implying that our new compounds do not have destabilizing effects on the molecular rhythm in contrast to KS15. Altogether, the results strongly suggest that **5d** and **2d** can be considered “circadian clock-enhancers” that strengthen the molecular circadian rhythmicity as well as E-box-mediated transcription evoked by the CLOCK:BMAL1 heterodimer through inhibition of CRY actions.

Nevertheless, it remains unclear how **5d** and **2d** reinforced cyclic PER2 protein accumulation in contrast to KS15. Both **5d** and **2d** maintained key structures required for binding to CRY proteins, which we previously identified in our structure–activity relationship study [18]. Indeed, **2d** and KS15 exhibited similar binding properties in SPR analyses with recombinant CRY proteins. Furthermore, **2d** showed no apparent isoform selectivity between CRY1 and CRY2 (Figure 4). It is plausible that these compounds may have distinct effects on a certain region/domain of CRY proteins residing outside their main binding site, thereby affecting post-translational regulation. In this context, it should be noted that the C-terminal domain, but not the binding pocket was responsible for the selectivity of isoform-specific CRY stabilizers, including KL101 and TH301 [7]. Therefore, **5d** and **2d** may have affected post-translational regulatory steps such as oligomerization with PER proteins, nuclear translocation, association with the CLOCK:BMAL1 heterodimer and/or inactivation/degradation, particularly, in a different way than KS15. The effects of these non-ethoxypropanoic acid-type inhibitors on the post-translational regulation of CRY proteins should be further examined to clarify the exact molecular mechanism(s) underlying their clock-enhancing activities.

## 3. Materials and Methods

### 3.1. Chemistry

Starting materials and reagents for chemical reactions were obtained from commercial suppliers unless otherwise stated. Organic solvents were dried over appropriate drying agents or distilled prior to use. We used Merck silica gel 60 F254 plates for thin-layer chromatography. Flash chromatography was performed with Biotage Isolera, and manual chromatography was conducted using Merck silica gel 60 (230–400 mesh). Nuclear magnetic resonance (NMR) spectra were acquired on a Bruker Avance-500 in deuterated chloroform (chloroform-*d*, CDCl_3_) or deuterated methanol (methanol-*d*, CD_3_OD); chemical shifts δ in parts per million (ppm) were measured relative to TMS with the residual solvent peak used as an internal reference, and J values were measured in Hertz. High-resolution mass spectrometry was conducted using Jeol JMS-700 at the Korea Basic Science Institute Daegu branch.

The analogs **5b**–**e** and **2b**–**e** were synthesized using previously reported procedures for **5a** and **2a** [18], or a slight modification of those procedures. NMR spectra for all new compounds **5b**–**e** and **2b**–**e** are available in the Supplementary Materials.

#### 3.1.1. Methyl 2-(3-acetylphenoxy)acetate (**4**)

Methyl chloroacetate (1.54 mL, 17.6 mmol) was added to a solution of 3-hydroxyacetophenone (**3**, 2.00 g, 1.47 mmol) and K_2_CO_3_ (6.10 g, 44.1 mmol) in DMF (50 mL). The reaction mixture was stirred overnight, and then filtered through a short pad of celite. Volatiles in the filtrate were removed under the reduced pressure, and the remaining residue was diluted with EtOAc. The diluted solution was washed with water and brine. The organic solution was dried over MgSO_4_, filtered, and concentrated. The residue was purified by SiO_2_ chromatography to afford **4** as a white solid (270 mg, 95% yield). Spectral data were well matched with previously reported data [18].

#### 3.1.2. Representative Procedure: Methyl 2-(3-(1-(((4-nitrobenzyl)oxy)imino)ethyl)phenoxy)acetate (**5d**)

Methyl 2-(3-acetylphenoxy)acetate (**4**, 1.08 g, 5.56 mmol) and *O*-(4-nitrobenzyl)hydroxylamine hydrochloride (1.37 g, 6.67 mmol) were dissolved in pyridine (24 mL). The reaction mixture was refluxed with stirring for 2 h at 120 °C. After cooled to rt, volatiles were removed under the reduced pressure. The residue was diluted with EtOAc, and then treated with 1*N*-HCl. The mixture was extracted with EtOAc, washed with water and brine. The organic solution was dried over MgSO_4_, filtered, and concentrated in vacuo. The residue was purified by SiO_2_ chromatography to afford the compound **5d** as a white solid (1.41 g, 71%).

#### 3.1.3. Characterization Data for **5b**–**e**

Methyl 2-(3-(1-(((3-trifluoromethylbenzyl)oxy)imino)ethyl)phenoxy)acetate (**5b**): colorless oil, 54% yield. Rf = 0.5 (EtOAc:n-hexane = 1:4). ^1^H NMR (500 MHz, Chloroform-*d*) δ 7.70 (m, 1H, Ar), 7.70–7.54 (m, 2H, Ar), 7.51 (t, *J* = 7.7 Hz, 1H, Ar), 7.33–7.25 (m, 3H, Ar), 6.94 (m, 1H, Ar), 5.30 (s, 2H, O-CH_2_-Ar), 4.68 (s, 2H, O-CH_2_-CO_2_Me), 3.71 (s, 3H, OCH_3_) 2.29 (s, 3H, CH_3_). ^13^C NMR (125 MHz, Chloroform-*d*) δ 169.4 (C=O), 157.9 (OAr), 155.2 (C=N), 139.3 (Ar), 138.1 (Ar), 138.1 (Ar), 131.4 (Ar), 130.8 (q, *J* = 32.3 Hz, C-CF_3_), 129.7 (Ar), 129.0 (Ar), 124.9 (q, *J* = 3.9 Hz, CH-C-CF_3_), 124.7 (q, *J* = 3.8 Hz, CH-C-CF_3_), 124.3 (q, *J* = 272.1 Hz, CF_3_), 119.9 (Ar), 115.5 (Ar), 112.6 (Ar), 75.5 (O-CH_2_-Ar), 65.5 (O-CH_2_-CO_2_Me), 52.4 (OCH_3_), 13.1 (CH_3_). HR-EI-MS *m*/*z* 381.1190 [M]^+^ (calculated for C_19_H_18_F_3_NO_4_ 381.1188). ATR-IR ν_max_ 2918, 2372, 2323, 1761 cm^−1^.

Methyl 2-(3-(1-(((4-methoxybenzyl)oxy)imino)ethyl)phenoxy)acetate (**5c**): White solid, 67% yield. Rf = 0.3 (EtOAc:n-hexane = 1:4). ^1^H NMR (500 MHz, Chloroform-*d*) δ 7.36 (d, *J* = 8.7 Hz, 2H, Ar), 7.29–7.24 (m, 3H, Ar), 6.92–6.87 (m, 3H, Ar), 5.16 (s, 2H, O-CH_2_-Ar), 4.66 (s, 2H, O-CH_2_-CO_2_Me), 3.81 (s, 3H, OCH_3_), 3.80 (s, 3H, OCH_3_), 2.21 (s, 3H, CH_3_). ^13^C NMR (125 MHz, Chloroform-*d*) δ 169.4 (C=O), 159.5 (OAr), 157.9 (OAr), 154.4 (C=N), 138.4 (Ar), 130.2 (Ar), 130.1 (2C, Ar), 129.6 (Ar), 119.9 (Ar), 115.3 (Ar), 113.9 (2C, Ar), 112.5 (Ar), 76.1 (O-CH_2_-Ar), 65.5 (O-CH_2_-CO_2_Me), 55.4 (OCH_3_), 52.4 (OCH_3_), 13.0 (CH_3_). HR-EI-MS *m*/*z* 343.1420 [M]^+^ (calculated for C_19_H_21_NO_5_ 343.1420). ATR-IR ν_max_ 2867, 2372, 2326, 1735 cm^−1^.

Methyl 2-(3-(1-(((4-nitrobenzyl)oxy)imino)ethyl)phenoxy)acetate (**5d**): White solid, 71% yield. Rf = 0.3 (EtOAc:n-hexane = 1:3). ^1^H NMR (500 MHz, Chloroform-*d*) δ 8.12 (d, 2H, *J* = 8.7 Hz, Ar), 7.45 (d, 2H, *J* = 8.8 Hz, Ar), 7.24–7.10 (m, 3H, Ar), 6.81 (m, 1H, Ar), 5.23 (s, 2H, O-CH_2_-Ar), 4.56 (s, 2H, O-CH_2_-CO_2_Me), 3.71 (s, 3H, OCH_3_), 2.18 (s, 3H, CH_3_). ^13^C NMR (125 MHz, Chloroform-*d*) δ 169.2 (C=O), 157.8 (OAr), 155.4 (C=N), 147.4 (Ar), 145.9 (Ar), 137.7 (Ar), 129.6 (Ar), 128.3 (2C, Ar), 123.6 (2C, Ar), 119.7 (Ar), 115.3 (Ar), 112.6 (Ar), 74.7 (O-CH_2_-Ar), 65.3 (O-CH_2_-CO_2_Me), 52.3 (OCH_3_), 12.9 (CH_3_). HR-EI-MS *m*/*z* 358.1165 [M]^+^ (calculated for C_18_H_18_N_2_O_6_ 358.1164). ATR-IR ν_max_ 2855, 2371, 2324, 1736 cm^−1^.

Methyl 2-(3-(1-(((4-methylsulfonylbenzyl)oxy)imino)ethyl)phenoxy)acetate (**5e**): White solid, 36% yield. Rf = 0.4 (EtOAc:n-hexane = 1:1). ^1^H NMR (500 MHz, Chloroform-*d*) δ 7.96 (d, *J* = 8.4 Hz, 2H, Ar), 7.61 (d, *J* = 8.4 Hz, 2H, Ar), 7.34–7.22 (m, 3H, Ar), 6.92 (m, 1H, Ar), 5.33 (s, 2H, O-CH_2_-Ar), 4.67 (s, 2H, O-CH_2_-CO_2_Me), 3.82 (s, 3H, OCH_3_), 3.08 (s, 3H, SO_2_CH_3_), 2.30 (s, 3H, CH_3_). ^13^C NMR (125 MHz, Chloroform-*d*) δ 169.2 (C=O), 157.8 (OAr), 155.4 (C=N), 144.8 (Ar), 139.7 (Ar), 137.8 (Ar), 129.6 (Ar), 128.4 (2C, Ar), 127.5 (2C, Ar), 119.8 (Ar), 115.3 (Ar), 112.7 (Ar), 74.9 (O-CH_2_-Ar), 65.3 (O-CH_2_-CO_2_Me), 52.3 (OCH_3_), 44.6 (SO_2_CH_3_), 13.0 (CH_3_). HR-EI-MS *m*/*z* 391.1090 [M]^+^ (calculated for C_19_H_21_NO_6_S 391.1092). ATR-IR ν_max_ 2926, 2371, 2324, 1755 cm^−1^.

#### 3.1.4. Representative Procedure: 2-(3-(1-(((4-nitrobenzyl)oxy)imino)ethyl)phenoxy)acetic acid (**2d**)

LiOH·H_2_O (281 mg, 6.70 mmol) was added to a solution of the compound **5d** (800 mg, 2.23 mmol) in THF (6 mL) and H_2_O (6 mL). The reaction mixture was stirred for 2 h, then acidified with 1*N*-HCl. The resulting mixture was extracted with EtOAc. The organic solution was dried over MgSO_4_, filtered, and concentrated in vacuo. The residue was purified by SiO_2_ chromatography to afford the compound **5d** as a white solid (543 mg, 71%).

#### 3.1.5. Characterization Data for **2b**–**e**

2-(3-(1-(((3-trifluoromethylbenzyl)oxy)imino)ethyl)phenoxy)acetic acid (**2b**): Colorless oil, 90% yield. Rf = 0.5 (MeOH:CH_2_Cl_2_ = 1:19). ^1^H NMR (500 MHz, Chloroform-*d*) δ 9.82 (br, 1H, CO_2_H), 7.57 (m, 1H, Ar), 7.51–7.43 (m, 2H, Ar), 7.37 (t, *J* = 7.7 Hz, 1H, Ar), 7.20–7.11 (m, 3H, Ar), 6.80 (m, 1H, Ar), 5.17 (s, 2H, O-CH_2_-Ar), 4.56 (s, 2H, O-CH_2_-CO_2_H), 2.15 (s, 3H, CH_3_). ^13^C NMR (125 MHz, Chloroform-*d*) δ 172.9 (C=O), 156.1 (OAr), 153.8 (C=N), 137.7 (Ar), 136.6 (Ar), 130.0 (Ar), 129.4 (q, *J* = 31.3 Hz, C-CF_3_), 128.3 (Ar), 127.5 (Ar), 123.4 (q, *J* = 3.8 Hz, CH-C-CF_3_), 123.2 (q, *J* = 3.8 Hz, CH-C-CF_3_), 122.8 (q, *J* = 271.3 Hz, CF_3_), 118.6 (Ar), 114.1 (Ar), 111.1 (Ar), 74.0 (O-CH_2_-Ar), 63.6 (OCH_3_), 11.6 (CH_3_). HR-EI-MS *m*/*z* 367.1031 [M]^+^ (calculated for C_18_H_16_F_3_NO_4_ 367.1029). ATR-IR ν_max_ 2936, 2372, 2324, 1735 cm^−1^.

2-(3-(1-(((4-methoxybenzyl)oxy)imino)ethyl)phenoxy)acetic acid (**2c**): White solid, 90% yield. Rf = 0.4 (EtOAc:n-hexane = 2:1). ^1^H NMR (500 MHz, Chloroform-*d*) δ 9.76 (br, 1H, CO_2_H), 7.37 (d, *J* = 8.6 Hz, 2H, Ar), 7.32–7.24 (m, 3H, Ar), 6.94–6.88 (m, 3H, Ar), 5.17 (s, 2H, O-CH_2_-Ar), 4.70 (s, 2H, O-CH_2_-CO_2_H), 3.81 (s, 3H, OCH_3_), 2.22 (s, 3H, CH_3_). ^13^C NMR (125 MHz, Chloroform-*d*) δ 174.1 (C=O), 159.4 (OAr), 157.4 (OAr), 154.4 (C=N), 138.4 (Ar), 130.0 (2C, Ar), 129.6 (Ar), 120.0 (Ar), 115.3 (Ar), 113.8 (2C, Ar), 112.3 (Ar), 76.1 (O-CH_2_-Ar), 64.8 (O-CH_2_-CO_2_H), 55.3 (OCH_3_), 13.0 (CH_3_). HR-EI-MS *m*/*z* 329.1263 [M]^+^ (calculated for C_18_H_19_NO_5_ 329.1267). ATR-IR ν_max_ 2906, 2372, 2326, 1733 cm^−^^1^.

2-(3-(1-(((4-nitrobenzyl)oxy)imino)ethyl)phenoxy)acetic acid (**2d**): White solid, 71% yield. Rf = 0.4 (MeOH:CH_2_Cl_2_ = 1:19). ^1^H NMR (500 MHz, Chloroform-*d*) δ 8.14 (d, *J* = 8.9 Hz, 2H, Ar), 7.47 (d, *J* = 8.7 Hz, 2H, Ar), 7.24–7.15 (m, 3H, Ar), 6.85 (m, 1H, Ar), 5.24 (s, 2H, O-CH_2_-Ar), 4.62 (s, 2H, O-CH_2_-CO_2_H), 2.21 (s, 3H, CH_3_). ^13^C NMR (125 MHz, Chloroform-*d*) δ 173.5 (C=O), 157.4 (OAr), 155.4 (C=N), 147.5 (Ar), 145.8 (Ar), 137.8 (Ar), 129.7 (Ar), 128.3 (2C, Ar), 123.7 (2C, Ar), 120.1 (Ar), 115.4 (Ar), 112.5 (Ar), 74.8(O-CH_2_-Ar), 64.8 (O-CH_2_-CO_2_H), 13.0 (CH_3_). HR-EI-MS *m*/*z* 344.1008 [M]^+^ (calculated for C_17_H_16_N_2_O_6_ 344.1005). ATR-IR ν_max_ 2910, 2372, 2324, 1739 cm^−^^1^.

2-(3-(1-(((4-methylsulfonylbenzyl)oxy)imino)ethyl)phenoxy)acetic acid (**2e**): White solid, 66% yield. Rf = 0.1 (EtOAc:n-hexane = 2:1). ^1^H NMR (500 MHz, Methanol-*d*_4_) δ 7.94 (d, *J* = 8.4 Hz, 2H, Ar), 7.65 (d, *J* = 8.1 Hz, 2H, Ar), 7.29–7.21 (m, 3H, Ar), 6.95 (m, 1H, Ar), 5.31 (s, 2H, O-CH_2_-Ar), 4.66 (s, 2H, O-CH_2_-CO_2_H), 3.10 (s, 3H, SO_2_CH_3_), 2.27 (s, 3H, CH_3_).^13^C NMR (125 MHz, Methanol-*d*_4_) δ 171.2 (C=O), 158.0 (OAr), 155.4 (C=N), 145.0 (Ar), 139.8 (Ar), 137.6 (Ar), 129.2 (Ar), 128.3 (2C, Ar), 127.1 (2C, Ar), 119.1 (Ar), 115.2 (Ar), 112.0 (Ar), 74.5 (O-CH_2_-Ar), 64.5 (O-CH_2_-CO_2_H), 43.0 (SO_2_CH_3_), 11.6 (CH_3_). HR-EI-MS *m*/*z* 377.0933 [M]^+^ (calculated for C_18_H_19_NO_6_S 377.0934). ATR-IR ν_max_ 2921, 2372, 2324, 1728 cm^−^^1^.

### 3.2. Cell Cultures and Luciferase Reporter Assay

Materials for cell culture were purchased from Thermo Fisher Scientific, unless otherwise stated. NIH3T3 and HEK293T cells were maintained in Dulbecco’s modified Eagle medium supplemented with 5% fetal bovine serum and 100 U/mL penicillin/streptomycin in a humidified atmosphere containing 5% CO_2_ at 37 °C. Plasmids and transfection experiments for luciferase reporter assay were described in our previous report [18]. For dose-response experiments, mouse NIH3T3 cells stably transfected with an E-box-LUC reporter [16,18] were plated in 12-well culture plates, treated as indicated for 36 h, and subjected to luciferase assay. For transient transfections, we seeded HEK293T cells in 12-well culture plates and used Lipofectamine® 3000 reagent to transfect cells with the E-box-LUC reporter and the indicated clock gene expression plasmids together with a thymidine kinase promoter-driven *Renilla* luciferase (pRL-TK) plasmids. We kept total DNA levels constant by adding an empty pcDNA3.1 plasmid. After 24 h of transfection, cells were treated with vehicle (0.1% DMSO) or chemical compounds at various concentrations for 36 h. Cell extracts were prepared by incubating cells in 0.3 mL reporter lysis buffer for 15 min at room temperature. Luciferase activity was measured with a commercial dual luciferase assay kit (Promega, Madison, WI, USA).

### 3.3. Cytochrome P450 Inhibition Assay

The inhibitory effects of KS15 and compound **5d** on the metabolism of five cytochrome P450 (P450) probe substrates were evaluated using previous methods with slight modifications [19,20]. The following P450 probe substrates were used: phenacetin (100 μM) for CYP1A2, tolbutamide (100 μM) for CYP2C9, omeprazole (20 μM) for CYP2C19, dextromethorphan (5 μM) for CYP2D6, and midazolam (5 μM) for CYP3A. Incubation mixtures containing pooled human liver microsomes (HLMs, XTreme 200, XenoTech, Lenexa, KS, USA), P450 probe substrates, and KS15 or compound **5d** (final concentrations: 0, 0.5, 2, 5, 20, and 50 μM) were preincubated for 5 min at 37 °C. After preincubation, the NADPH-generating system (including 3.3 mM G6P, 1.3 mM β-NADP^+^, 3.3 mM MgCl_2_, and 1.0 U/mL G6PDH) was added to the incubation mixtures, and then incubated for 10 min at 37 °C. The reaction was stopped by adding 50 μL of 7 nM trimipramine (internal standard, IS) in ice-cold acetonitrile. After centrifugation at 18,000× *g* (5 min, 4 °C), supernatant aliquots were analyzed by liquid chromatography-tandem mass spectrometry (LC-MS/MS). All microsomal incubations were conducted in triplicate.

All metabolites and IS were separated on a Kinetex C18 column (100 mm × 2.1 mm, 2.6 μm ID., Phenomenex, Torrance, CA, USA) and analyzed using an LC-MS/MS system (LCMS 8060, Shimadzu, Tokyo, Japan) equipped with an electrospray ionization (ESI) interface. The mobile phase solvent A was 0.1% formic acid in water, and solvent B was 0.1% formic acid in acetonitrile. Gradient separation was performed as follows: 0–7 min (10–90% B) and 7.1–10 min (10% B). The flow rate was 0.2 mL/min, and the run time was 10 min for each sample. Electrospray ionization was conducted in the positive ion mode at 4 kV. Quantitation was performed in the selected reaction monitoring (SRM) of the [M+H]^+^ ion and the related product ion for each metabolite and IS: acetaminophen (*m*/*z* 152 > 110, collision energy (CE) 25 eV), 4-hydroxytolbutamide (*m*/*z* 287 > 89, CE 60 eV), 5-hydroxyomeprazole (*m*/*z* 362 > 214, CE 10 eV), dextromethorphan (*m*/*z* 258 > 157, CE 35 eV), 1′-hydroxymidazolam (*m*/*z* 342 > 203, CE 25 eV), and IS (*m*/*z* 295 > 100, CE 17 eV).

### 3.4. Metabolic Stability Test

The metabolic stability of KS15, **5d**, or **2d** was evaluated using pooled HLMs. Pooled HLMs (1.0 mg/mL), a potassium phosphate buffer, and a test compound (1 μM) were pre-incubated (37 °C, 5 min). To initiate the reaction, the NADPH generating system was added to the samples and incubated for specific time points (0, 5, 15, 20, 45, and 60 min). Then, aliquots were taken and reactions terminated in acetonitrile containing IS. After centrifugation, supernatants were analyzed using LC-MS/MS to determine KS15, **5d**, or **2d** concentrations in samples. Analyte separation was performed using a Kinetex C18 column (100 mm × 2.1 mm, 2.6 μm ID., Phenomenex). The mobile phase solvent A was 0.1% formic acid in water, and solvent B was 0.1% formic acid in acetonitrile. Gradient separation was performed as follows: 0–3 min (10–90% B) and 3.1–8 min (10% B). The flow rate was 0.2 mL/min, and the run time was 8 min for each sample. Electrospray ionization was conducted in positive ion mode at 4 kV. Optimum operating conditions were as follows: vaporizer temperature, 300 °C; capillary temperature, 250 °C; and collision gas (argon) pressure 17 kPa. Quantitation was performed in the SRM of the [M+H]^+^ ion and the related product ion for each test compound and IS: KS-15 (*m*/*z* 420 > 169, CE 30 eV), **5d** (*m*/*z* 359 > 206, CE 20 eV), **2d** (*m*/*z* 345 > 192, CE 22 eV), and IS (*m*/*z* 295 > 100, CE 17 eV).

### 3.5. Pharmacokinetics

For in vivo pharmacokinetic studies, Sprague Dawley (SD) male rats (weighing 270–280 g) were purchased from Orient Bio Inc. (Seongnam, Korea). Experimental protocols involving animal use were carefully reviewed and approved by the Institutional Animal Care and Use Committee at Gachon University. Prior to studies, animals fasted for 18 h, but with free access to water. Compound **5d** or **2d** was dissolved in a dosing vehicle consisting of PEG400, normal saline, and dimethylsulfoxide in a ratio of 6:3:1 (*v*/*v*/*v*%) to make a 2 mg/mL dosing solution. This was intravenously bolus-injected into the right femoral vein at a volume of 1 mL/kg (2 mg/kg) for intravenous administration or intragastrically injected using an oral zone at a volume of 2.5 mL/kg (5 mg/kg) for p.o. administration. Blood samples (approximately 100 μL each) were collected in heparinized tubes via the right femoral artery at 0, 1, 5, 15, 30, 60, 120, 240, 360, 480, 1440 min after intravenous administration, and 0, 5, 15, 30, 60, 90, 120, 240, 360, 480, 1440 min after p.o. administration.

To determine drug concentration levels in plasma, the Agilent 1290 UHPLC-DAD system with Polar RP column (3 μm, 150 mm × 2 mm ID., Phenomenex) was used for chromatographic separations. The mobile phase was composed of 0.1% formic acid in acetonitrile and 0.1% formic acid in DDW in a ratio of 40:60 and was isocratically delivered at a flow rate of 0.2 mL/min. The total run time was 4.4 min for samples which were monitored at 260 nm. Working standard solutions of compound **2d** were serially prepared in methanol in the 50–10,000 ng/mL concentration range, and 10 μL aliquots were added to 90 μL blank plasma to prepare calibration standards. For sample preparation, 100 μL aliquots of plasma samples or calibration standards were transferred to microcentrifuge tubes and mixed by vortexing vigorously for 5 min. They were then centrifuged at 14,000 rpm for 10 min at 4 °C. Supernatants were transferred to fresh analysis vials and a 2 μL aliquot injected onto the UHPLC-DAD system.

### 3.6. Surface Plasmon Resonance

We used a ProteOn XPR36 system (Bio-Rad, Hercules, CA, USA) equipped with a HTE sensor chip (high capacity tris-NTA for histidine-tagged small molecule capture, Bio-Rad) for surface plasmon resonance analyses. Purified target proteins (6xHis- and GST-tagged hCRY1 and hCRY2) were prepared by a baculovirus expression system (MyBioSource, San Diego, CA, USA). Phosphate buffered saline (PBS) plus 0.005% Tween 20 was used as the running buffer. The 6xHis-hCRY1 and 6xHis-hCRY2 proteins were immobilized onto separate channels of a HTE chip by injecting 210 μL protein sample (2.0 μg/mL) into flow cells (30 μL/min) to allow saturation. Serially diluted compound **2d** samples (50–800 μM) were prepared in 4% DMSO in TBS-T buffer. Real-time binding kinetics analysis was performed by injecting 100 μL sample aliquots into the channels of the HTE chip for 1 min at 100 μL/min. Then PBS-T buffer (100 μL/min) was flushed over the chip for 200 s to dissociate bound analytes. Recorded resonance units (RU) were normalized and plotted against chemical concentration. We used ProteOn Manager v.3.1.0.6 software for recording, plotting, and analyzing binding curves.

### 3.7. Real-Time Bioluminescence Monitoring

For real-time bioluminescence monitoring, we used mouse embryonic fibroblast (MEF) cultures established from Per2-LUC-KI mice [16,21]. To examine the possible effects of CRYs inhibitors on cyclic PER2 accumulation, Per2-LUC-KI MEFs were plated in 35 mm dishes, grown for 24 h, and synchronized with 200 nM DEX for 2 h. The medium was then replaced with a recording medium containing 0.1 mM D-luciferin (Promega) along with vehicle (0.1% DMSO), KS15 (20 μM), **5d** (20 μM), or **2d** (20 μM). Light emission for 2 min at intervals of 20 min was integrated using a dish-type wheeled luminometer (Kronos-Dio, ATTO Cooperation, Tokyo, Japan). Background-subtracted bioluminescence profiles were analyzed using Cosinor analysis software (available at http://www.circadian.org, last accessed on 19 May 2021).

### 3.8. Statistical Evaluation

The EC_50_ values for luciferase reporter assay were calculated based on a logistic four-parametric equation using the mean values of intrinsic activation (SigmaPlot 10.0; Systat Software Inc., San Jose, CA, USA). The IC_50_ and t_1/2_ values for the cytochrome P450 inhibition assay and metabolic stability test were calculated by WinNonlin software (Pharsight, Mountain View, CA, USA). Pharmacokinetic parameters were calculated using non-compartmental analysis (WinNonlin v.8.3; Pharsight, Mountain View, CA, USA). Kinetic and equilibrium constants for SPR analysis were obtained by global fitting of the Langmuir 1:1 bimolecular kinetic model (ProteOn Manager v.3.1.0.6, Bio-Rad, Hercules, CA, USA). Circadian molecular clock-enhancing activities were expressed as means ± SE and statistically evaluated by Student’s *t*-test or one-way analysis of variance (ANOVA) followed by Newman–Keuls multiple comparison test for post hoc comparisons. The significance level for the analyses was set at *p* < 0.05.

## 4. Conclusions

Bioisosteric replacement is a useful and prevalent strategy for drug design and molecular modification in medicinal chemistry. Years of cumulative data proved that rational design with such a strategy for modifying lead compounds provided better candidates with improved potency, selectivity, and drug-likeness [22,23,24]. In this context, we undertook synthesis and evaluation of non-ethoxypropanoic acid analogs of KS15 by bioisosteric replacement. Among the synthesized, compound **5d** and its hydrolyzed product **2d** were potent and effective enhancers on the E-box mediated transcription. Compounds **5d** and **2d** also exhibited desirable metabolic and pharmacokinetic profiles as promising drug candidates. Furthermore, compound **2d** directly binds to CRY1 and 2 in surface plasmon resonance analyses, suggesting that the pharmacological target of **2d** is CRYs. Compounds **5d** and **2d** inhibited the negative feedback actions of CRY on the CLOCK:BMAL1 heterodimer, which also suggested that their molecular target is CRYs. Most importantly, compounds **5d** and **2d** exhibited significant enough effects on molecular circadian rhythmicity to be considered circadian clock-enhancers, which are distinct from the KS15 possessing ethoxypropanoic acid moiety. Taken altogether, novel cryptochrome inhibitors with circadian clock-enhancing activity have been developed by bioisosteric replacement.

## Figures and Tables

**Table 1:** Effect of the synthesized derivatives on the E-box-LUC reporter activities.

Entry	Compound	Max (%) ^1^	EC_50_ (μM)
1	**2a**	164.8	1.54
2	**5a**	149.1	n/d ^2^
3	**2b**	130.7	n/d
4	**5b**	171.5	n/d
5	**2c**	182.1	9.88
6	**5c**	151.8	6.32
7	**2d**	161.3	10.17
8	**5d**	193.0	6.44
9	**2e**	98.7	n/d
10	**5e**	104.7	n/d

^1^ Maximum responses expressed as a percentage of the vehicle-treated group. ^2^ EC_50_ values were not determined within the tested dosages.

**Table 2:** Inhibitory effects of KS15 and **5d** on specific cytochrome P450 (P450) activities in human liver microsomes.

P450	Enzyme Activity	IC_50_ (μM)	
		KS15	5d
1A2	Phenacetin *O*-deethylation	14.9	>50
2C9	Tolbutamide 4-methylhydroxylation	9.1	>50
2C19	Omeprazole hydroxylation	20.2	>50
2D6	Dextromethorphan *O*-demethylation	23.1	>50
3A	Midazolam 1’-hydroxylation	24.0	>50

**Table 3:** Pharmacokinetic parameters of **5d** and **2d** following PO and IV administrations.

Parameter	Intravenous (2 mg/kg)		Oral (5 mg/kg)	
	5d	2d	5d	2d
AUC_last_ (μg/mL·min)	2891.2 ± 465.0	3885.9 ± 278.5	9471.5 ± 1281.7	10,204.7 ± 1308.5
AUC_infinite_ (μg/mL·min)	3268.2 ± 600.3	4290.6 ± 398.7	n/a	11,069.2 ± 1130.5
t_1/2_ (min)	467.1 ± 61.3	433.0 ± 58.0	n/a	391.2 ± 47.6
C_max_ (μg/mL)	-	-	13.5 ± 4.7	14.6 ± 5.1
t_max_ (min)	-	-	420.0 ± 69.3	160.0 ± 69.2
CL (mL/min/kg)	0.6 ± 0.1	0.5 ± 0.0	-	-
MRT (min)	588.5 ± 75.6	557.1 ± 73.7	-	-
Vss (mL/kg)	349.8 ± 34.9	259.7 ± 25.8	-	-
F (%)	-	-	> 99	> 99

## Data Availability

The data presented in this study are available in the article and Supplementary Material.

## Supplementary Materials

The Supplementary Material for this article is available online at https://www.mdpi.com/article/10.3390/ph14060496/s1, including 1H and 13C NMR spectra.
