# Supplementary material for: Development of Non-Ethoxypropanoic Acid Type Cryptochrome Inhibitors with Circadian Molecular Clock-Enhancing Activity by Bioisosteric Replacement
Source: Pharmaceuticals (Basel). 2021 May 24;14(6):496. doi: 10.3390/ph14060496 (PMC8225008; doi:10.3390/ph14060496)

<Supplementary Materials>

Development of Non-Ethoxypropanoic Acid Type Cryptochrome Inhibitors with Circadian Molecular Clock-Enhancing Activity by Bioisosteric Replacement

Yong Uk Jeong [1,2,†], Hyo-Eon Jin [3,†], Hye Young Lim[4], Goyeong Choi [1], Hansol Joo [1,2], Bohun Kang [1,2], Ga-Hyun Lee [1], Kwang-Hyeon Liu [1], Han-Joo Maeng [5], Sooyoung Chung [6], Gi Hoon Son [4,*] and Jong-Wha Jung [1,2,*]

1 Research Institute of Pharmaceutical Sciences, College of Pharmacy, Kyungpook National University, Daegu 41566, Korea; nemo946@naver.com (Y.U.J.); qlgudtmfkqmf@naver.com (G.C.); wnthf0518@naver.com (H.J.); bohun0609@gmail.com (B.K.); lgh2710@gmail.com (G.-H.L.); dstlkh@knu.ac.kr (K.L.); jungj@knu.ac.kr (J.-W.J.)

2 Vessel-Organ Interaction Research Center, Kyungpook National University, Daegu 41566, Korea

3 College of Pharmacy, Ajou University, Suwon 16499, Korea; hjin@ajou.ac.kr (H.-E.J.)

4 Department of Biomedical Sciences, College of Medicine, Korea University, Seoul 02841, Korea; yeppi0905@naver.com (H.Y.L.); songh@korea.ac.kr (G.H.S.)

5 College of Pharmacy, Gachon University, Incheon 21936, Korea; hjmaeng@gachon.ac.kr

6 Department of Brain and Cognitive Sciences, Scranton College, Ewha Womans University, Seoul 03760, Korea; csooy@ewha.ac.kr (S.C.)

* Correspondence: songh@korea.ac.kr (G.H.S.); jungj@knu.ac.kr (J.-W.J.); Tel.: +82-2-2286-1147 (G.H.S.); +82-53-950-8578 (J.-W.J.)

† These authors equally contributed to the work.

^{1}H and ^{13}C NMR spectra for compound 5b-e and 2b-e

Compound 5b

^{1}H-NMR

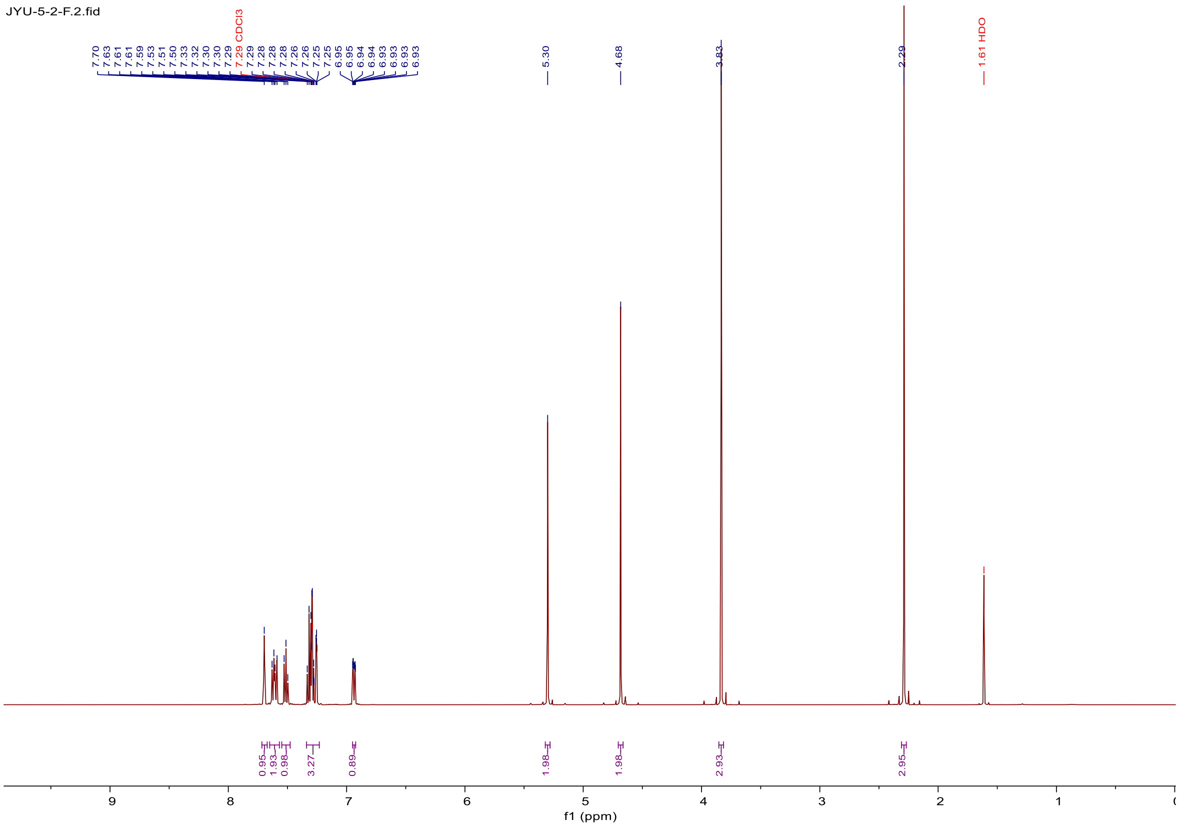

^{13}C-NMR

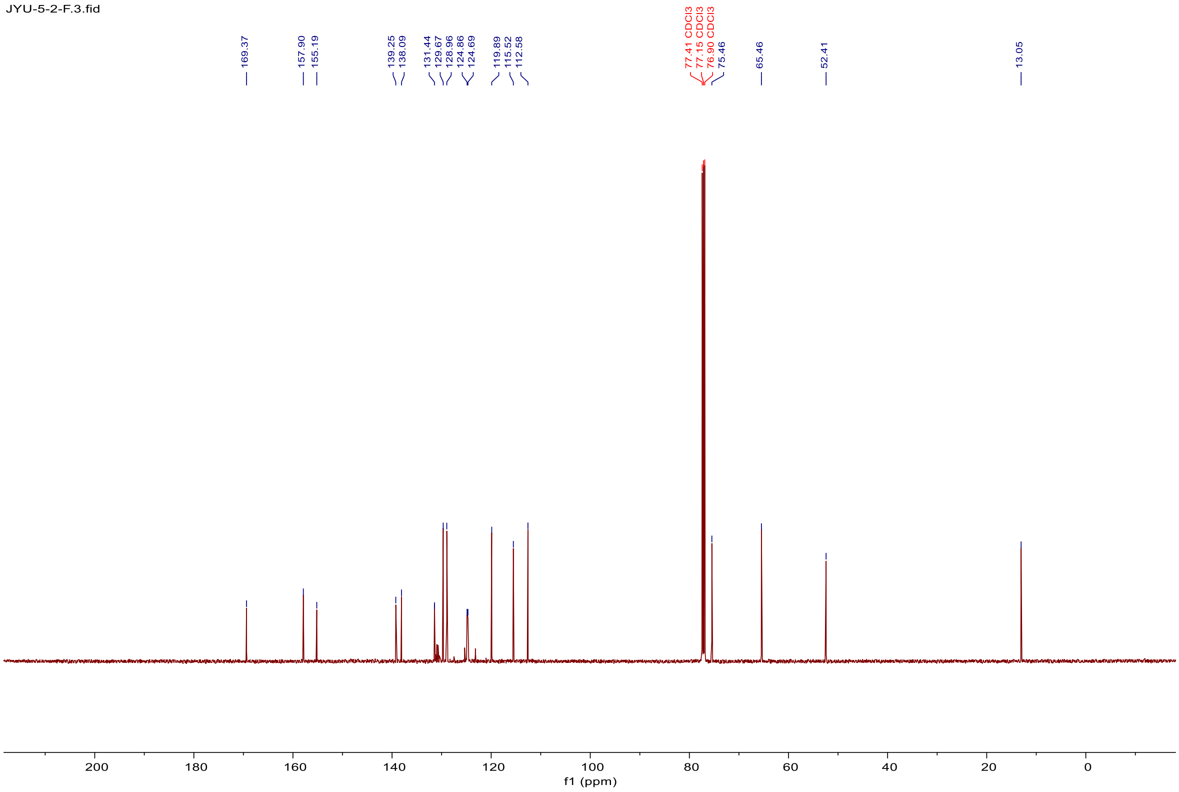

Compound 5c

^{1}H-NMR

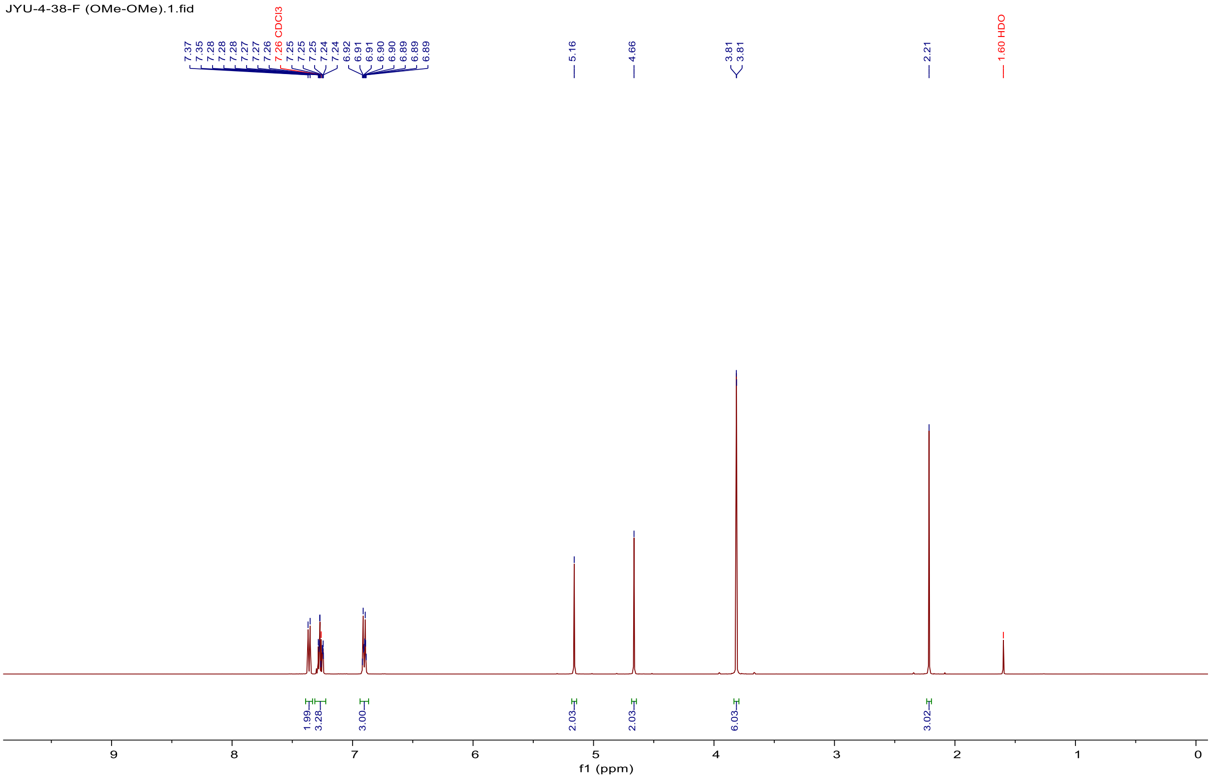

^{13}C-NMR

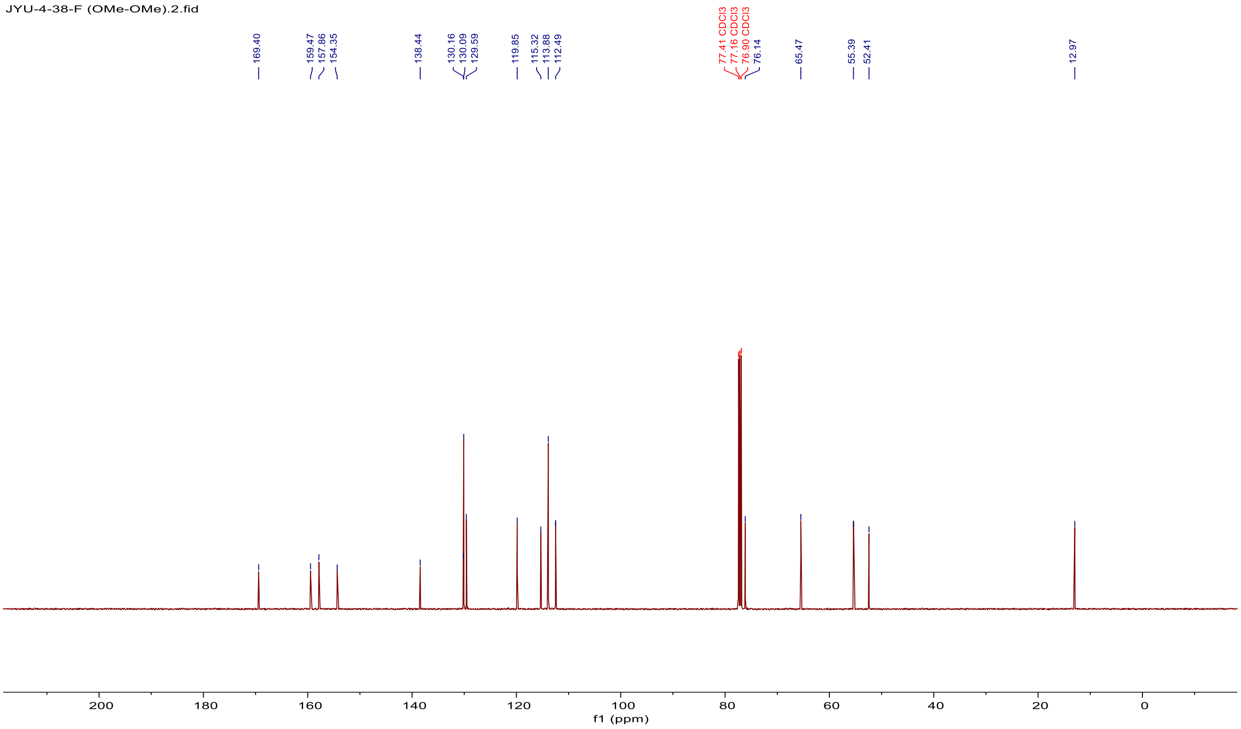

Compound 5d

^{1}H-NMR

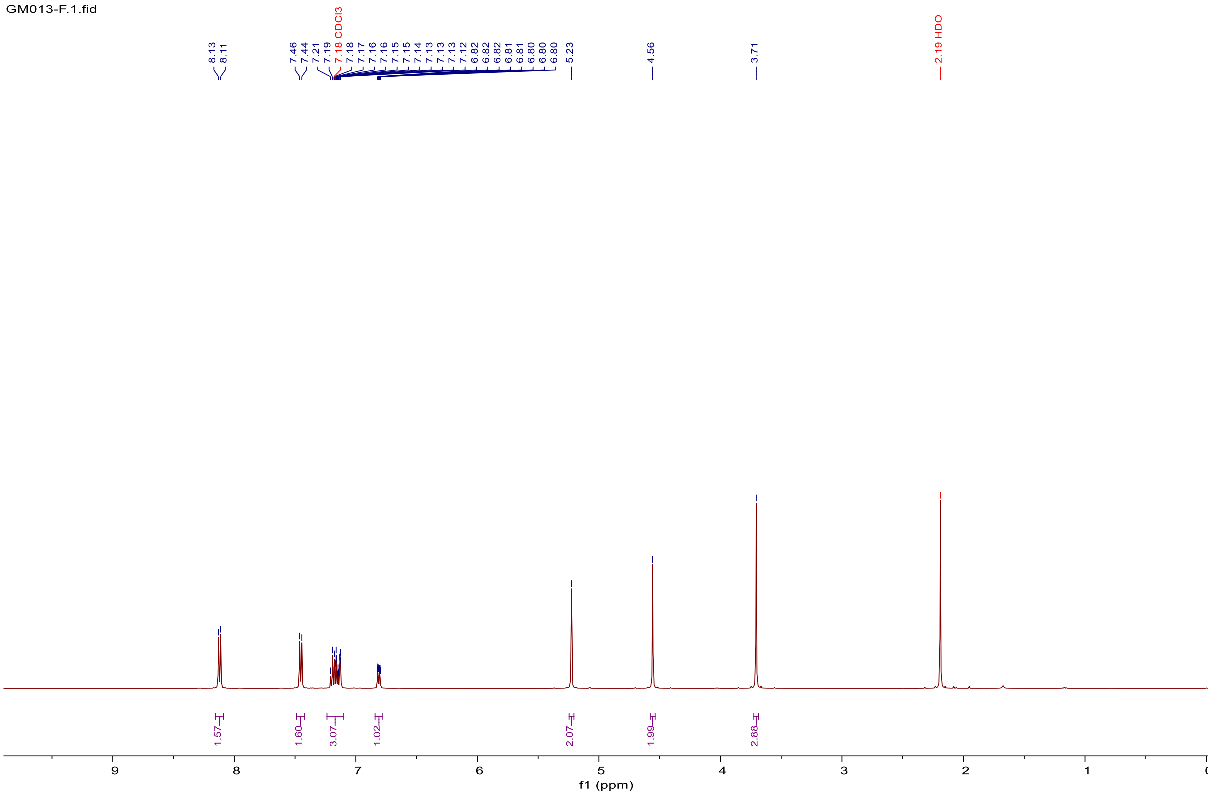

^{13}C-NMR

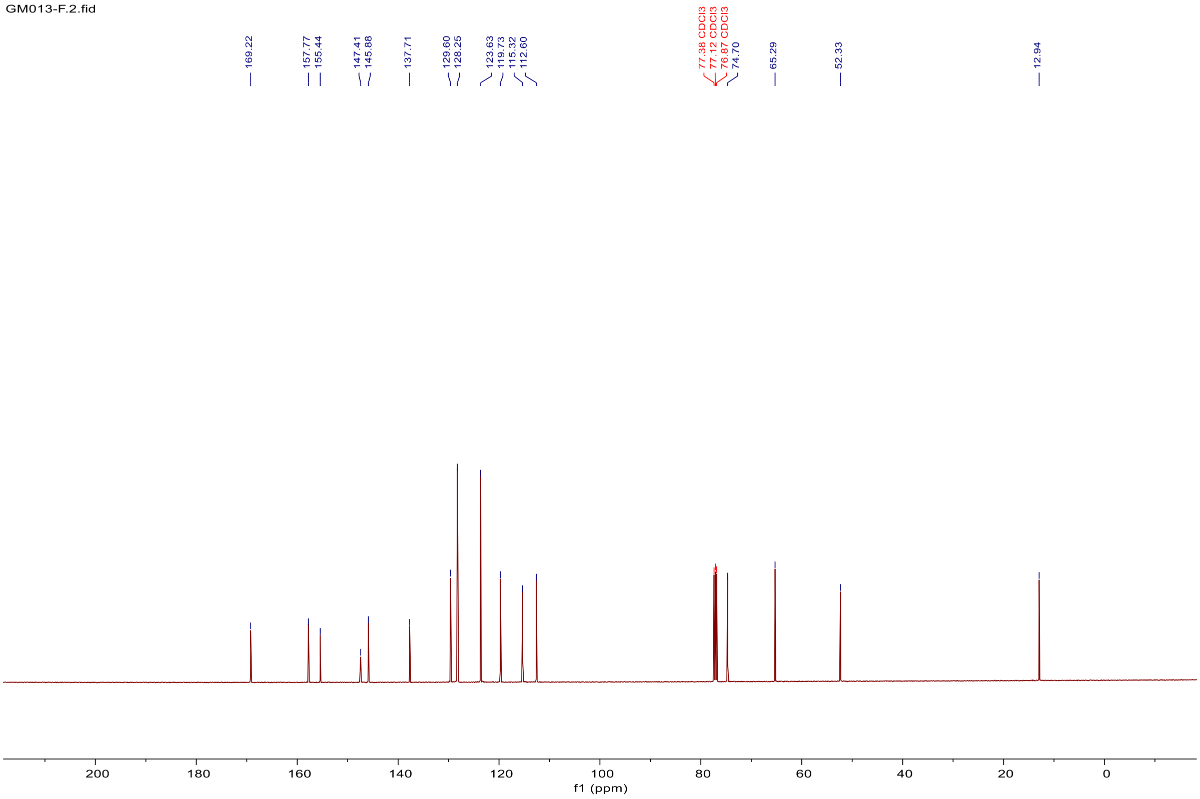

Compound 5e

¹H-NMR

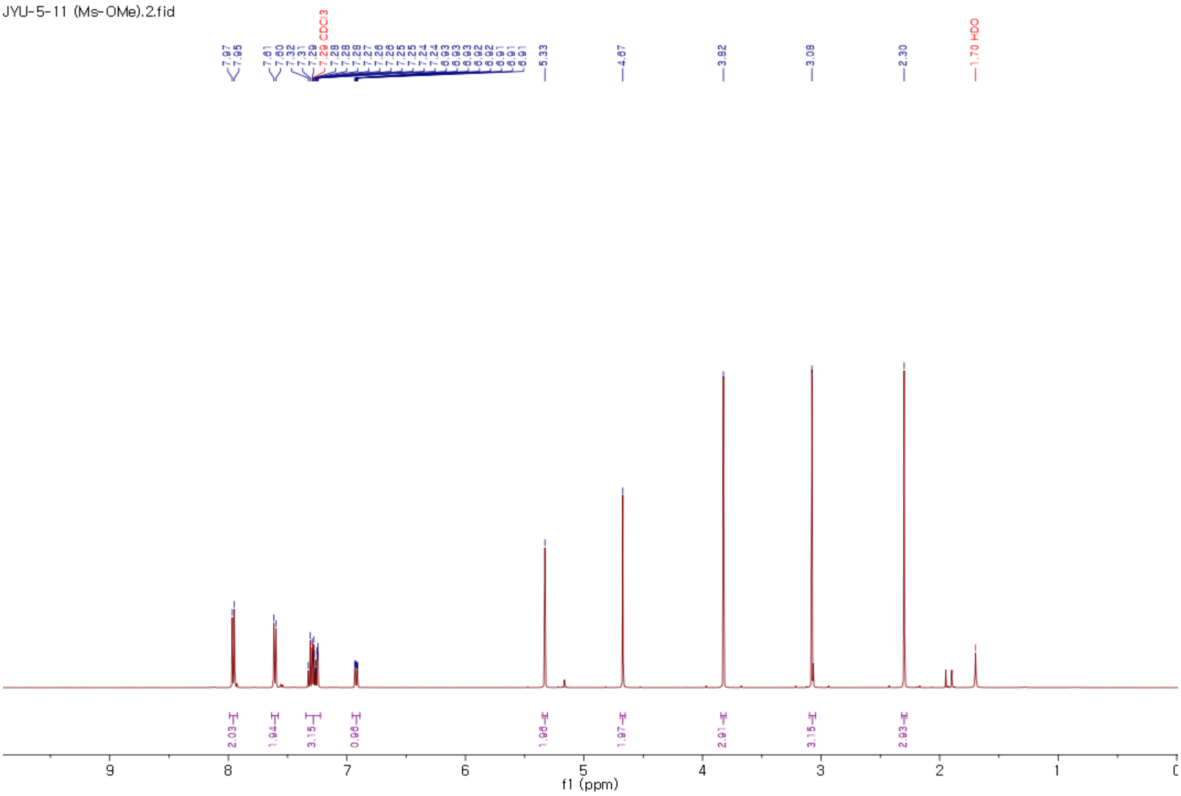

¹³C-NMR

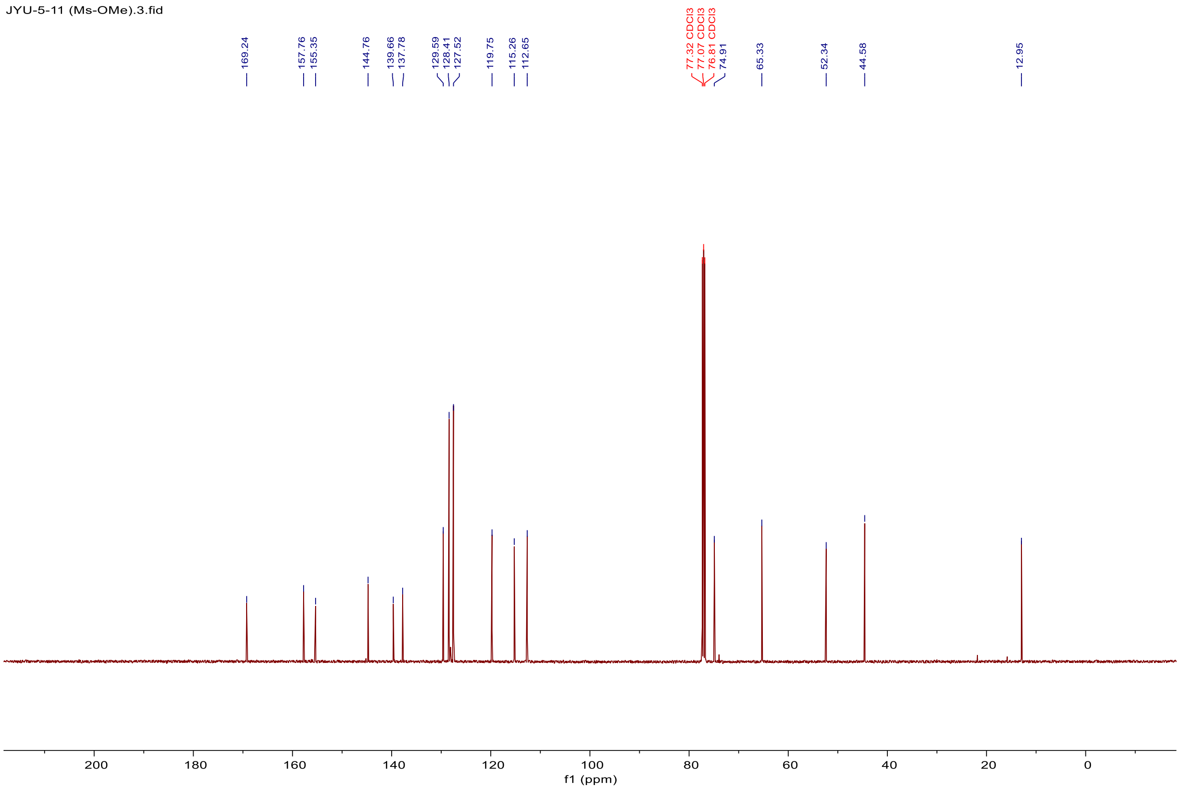

Compound 2b

^{1}H-NMR

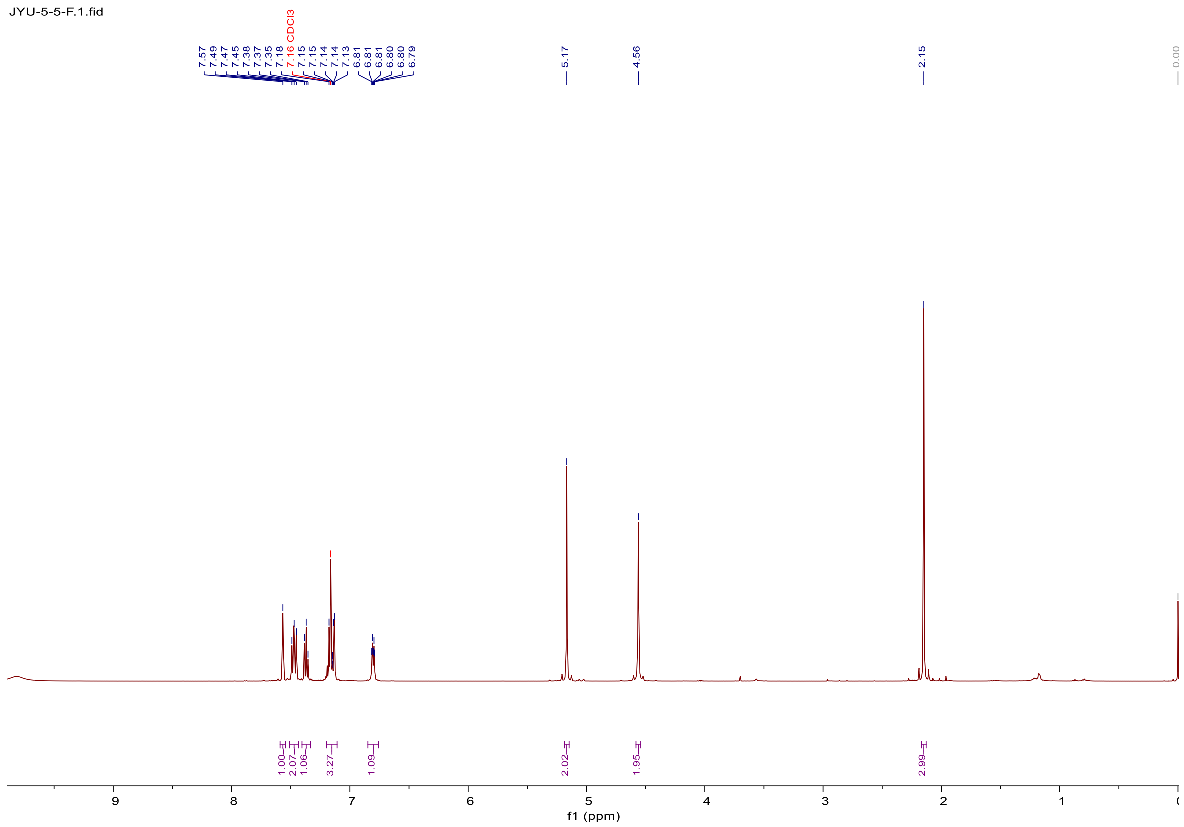

^{13}C-NMR

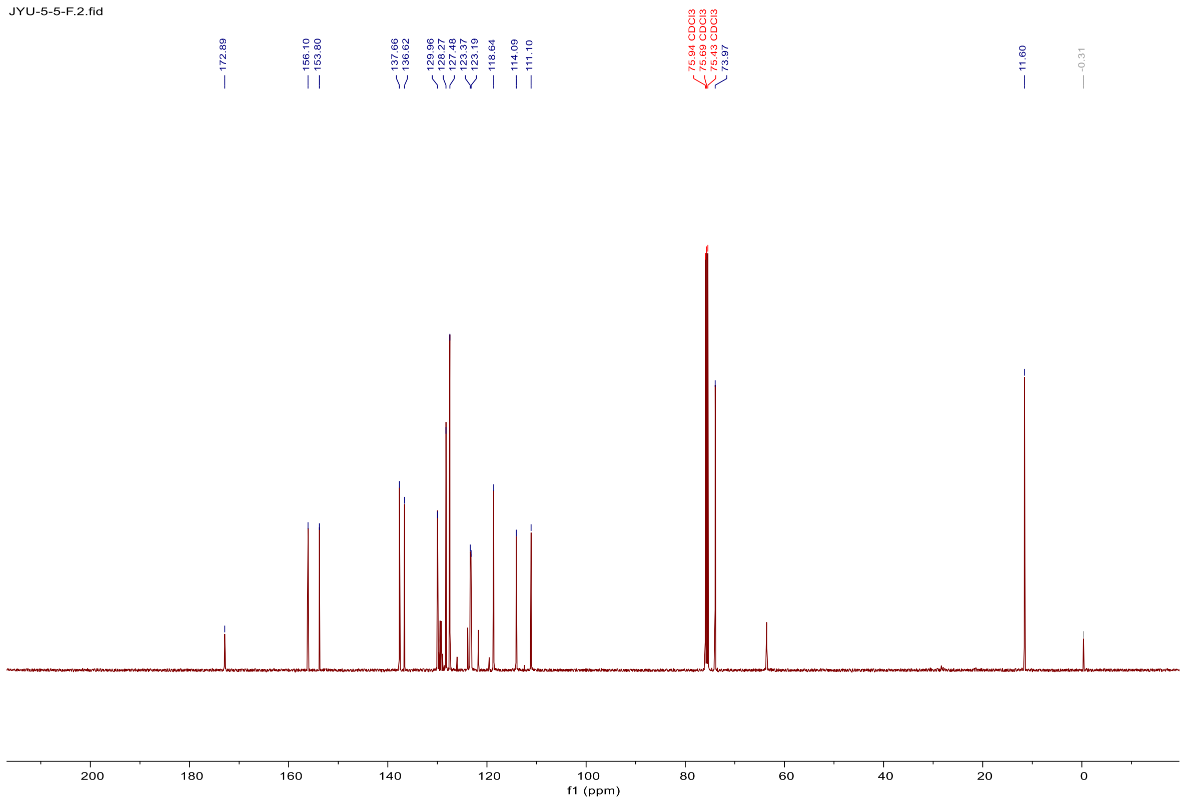

Compound 2c

^{1}H-NMR

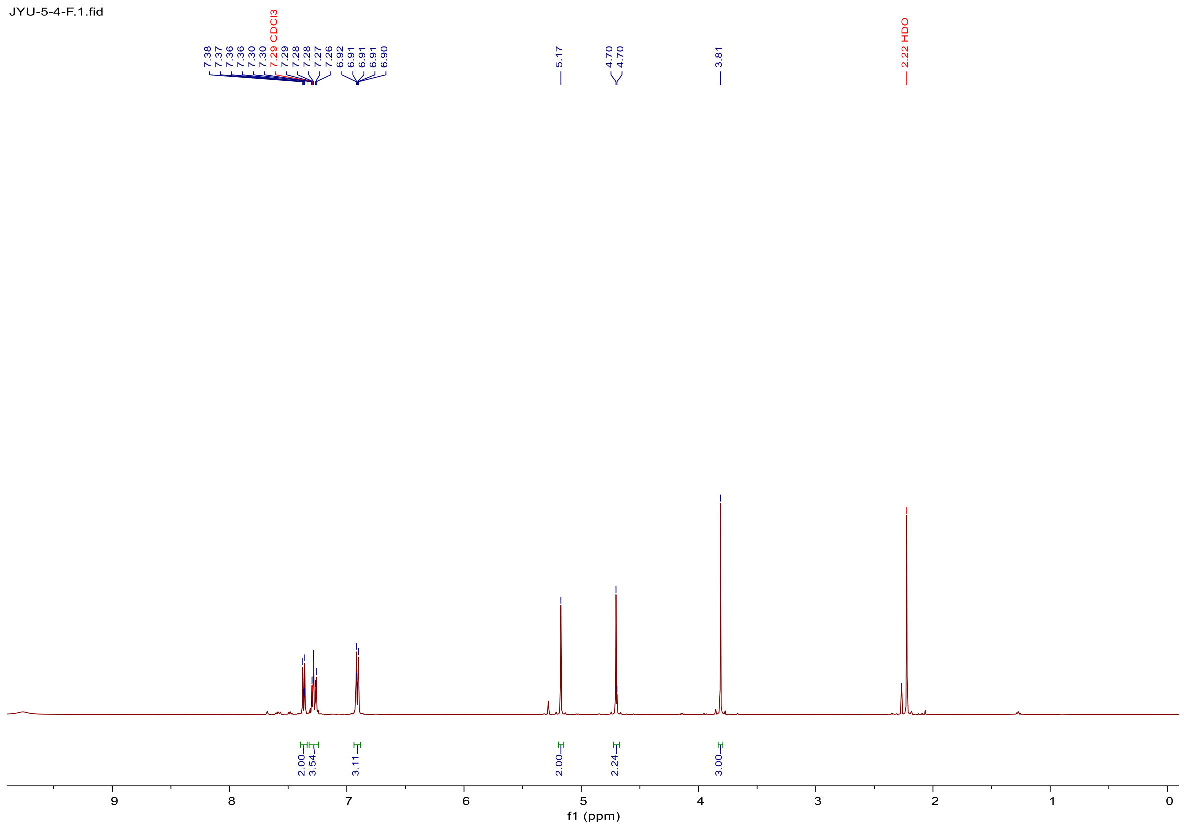

^{13}C-NMR

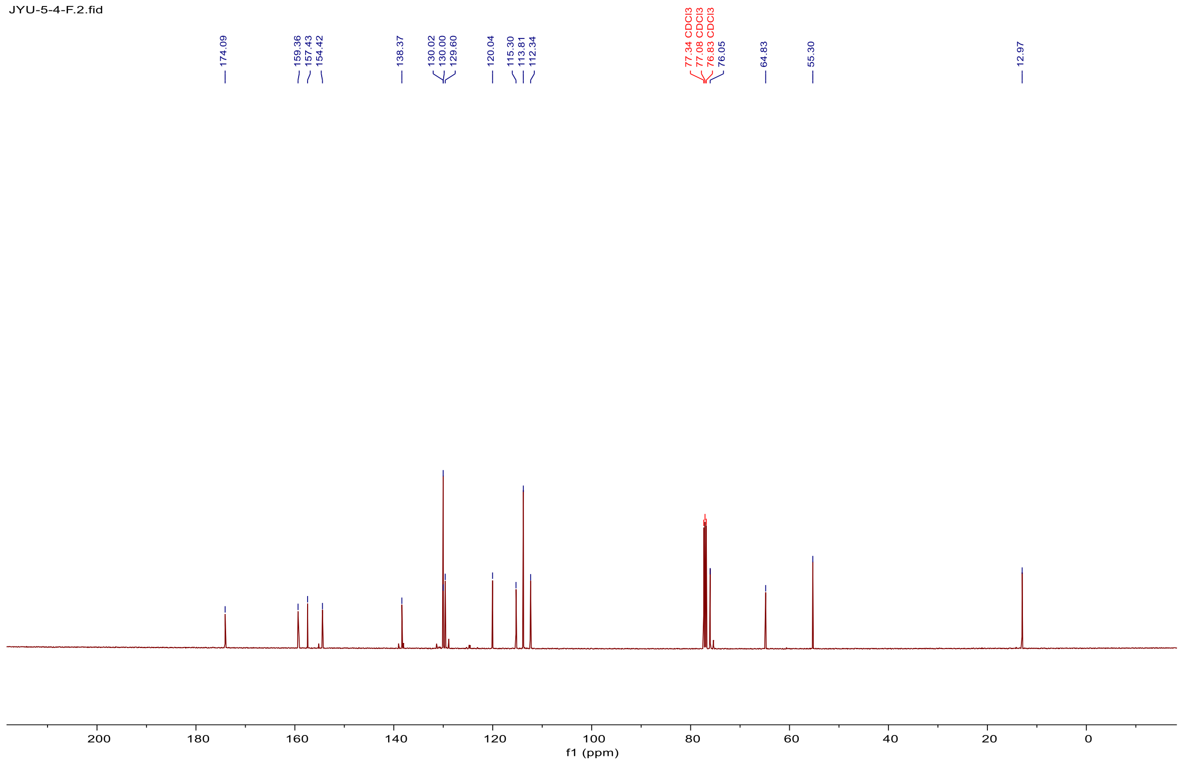

Compound 2d

^{1}H-NMR

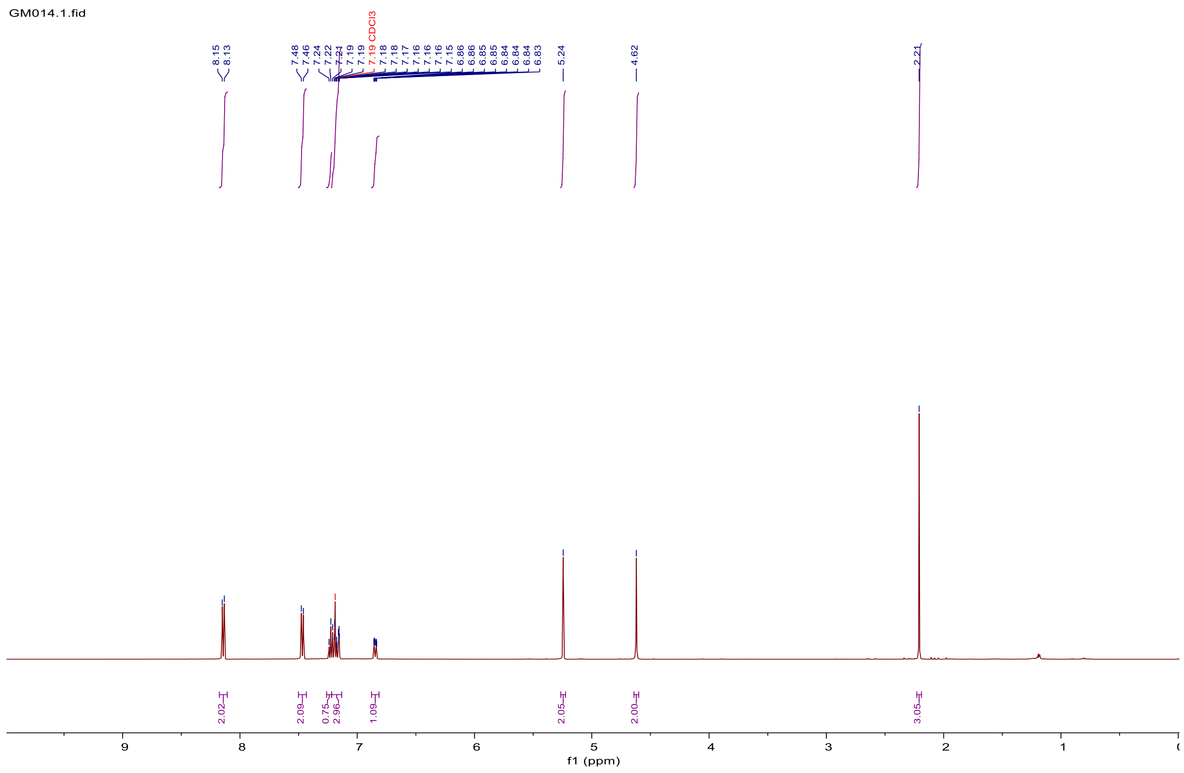

^{13}C-NMR

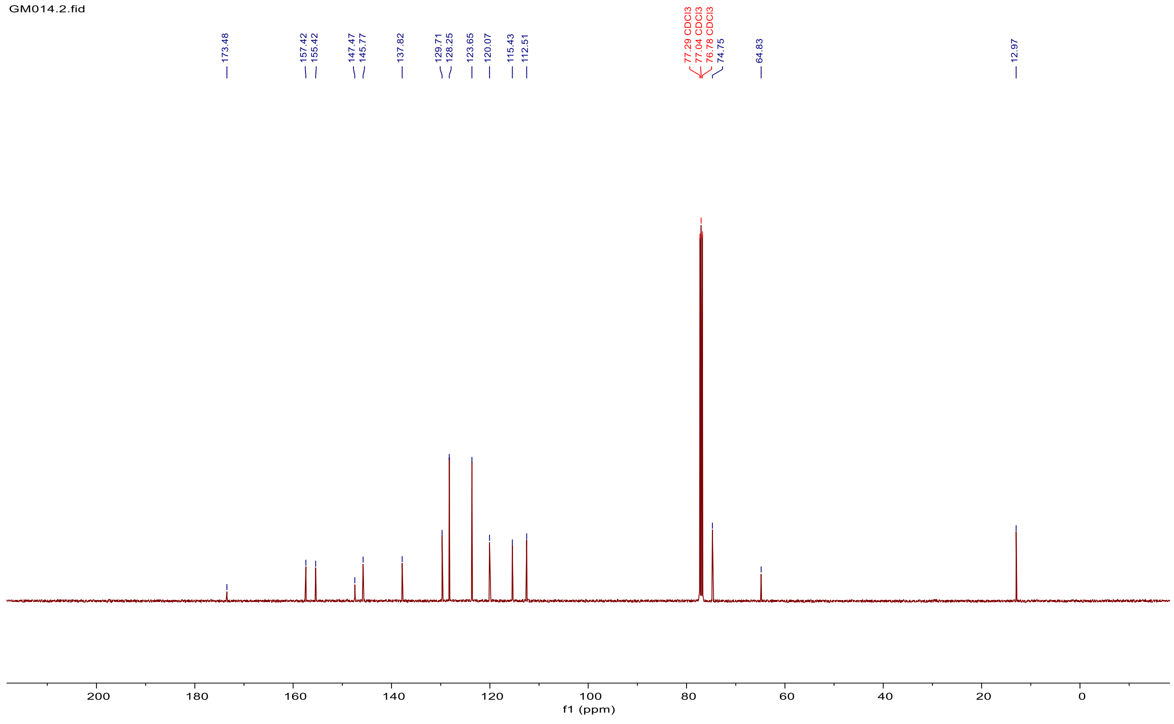

Compound 2e

^{1}H-NMR

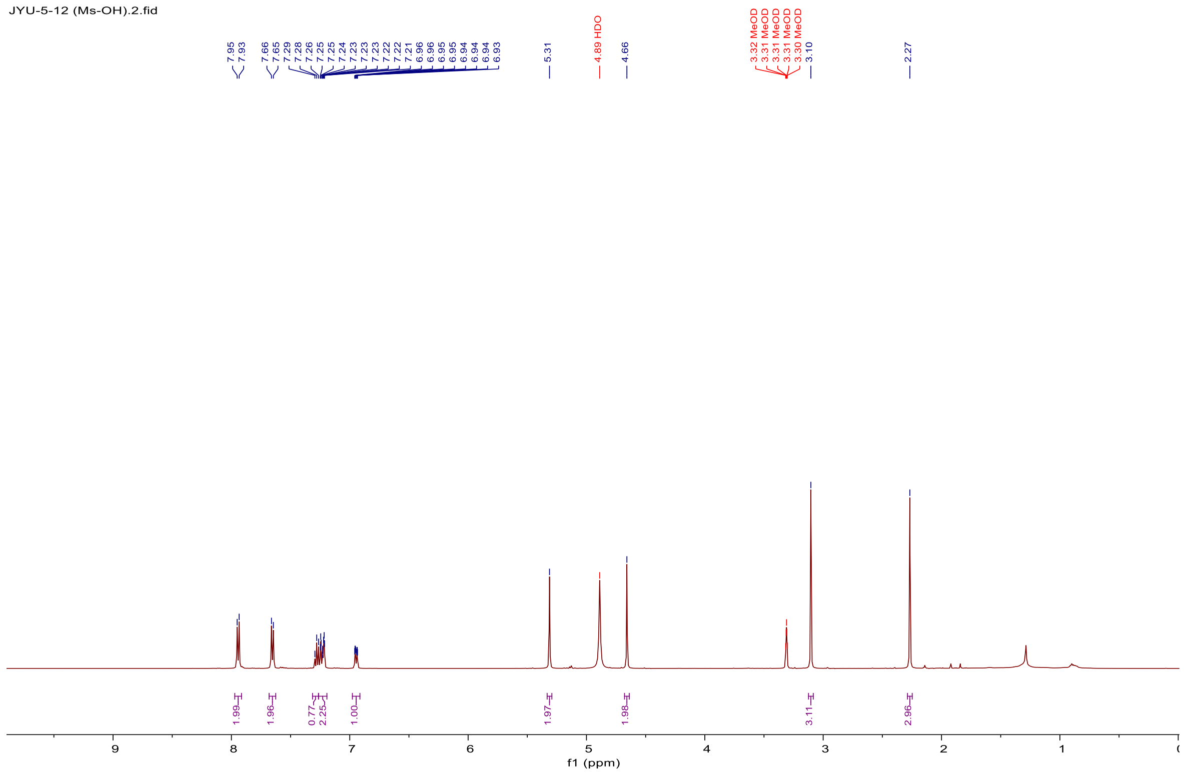

^{13}C-NMR

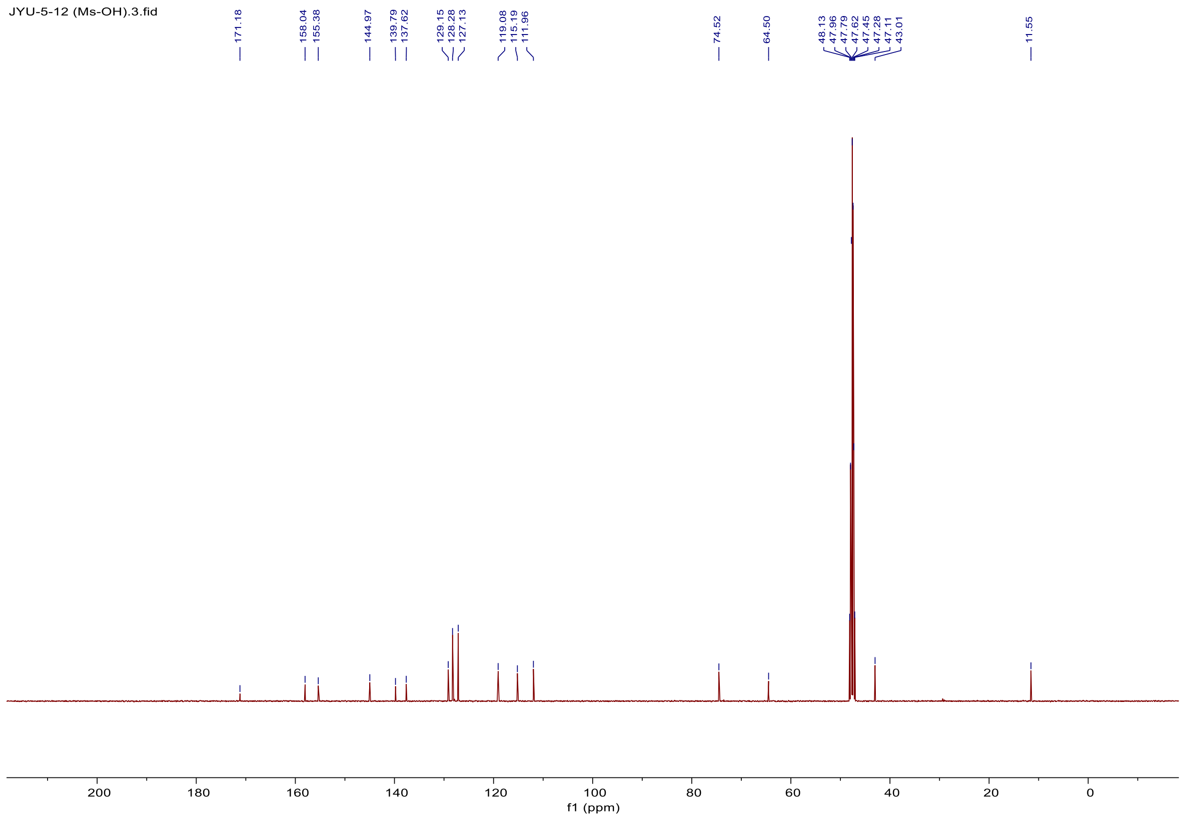

Supplement: Supplementary file 1 [file pharmaceuticals-14-00496-s001.zip › pharmaceuticals-1202639-supplementary.pdf]
